# Health Care Utilization and Out-of-Pocket Payments among Elderly with Cognitive Frailty in Malaysia

**DOI:** 10.3390/ijerph19063361

**Published:** 2022-03-13

**Authors:** Ahmed Abdelmajed Alkhodary, Syed Mohamed Aljunid, Aniza Ismail, Amrizal Muhammad Nur, Suzana Shahar

**Affiliations:** 1 International Centre for Casemix and Clinical Coding, Faculty of Medicine, National University of Malaysia, Kuala Lumpur 56000, Malaysia; akh77@live.com (A.A.A.); saljunid@gmail.com (S.M.A.); amrizal65@gmail.com (A.M.N.); 2 Ministry of Health, Department of Analysis and Decision Support, Gaza Governorates, Al Remal, Gaza 0101, Palestine; 3 Department of Health Policy and Management, Faculty of Public Health, Kuwait University, Kuwait 5969, Kuwait; 4 Department of Community Health, Faculty of Medicine, Universiti Kebangsaan Malaysia, Jalan Yaakob Latiff, Bandar Tun Razak, Cheras, Kuala Lumpur 56000, Malaysia; 5 Program of Dietetics, Faculty of Health Science, National University of Malaysia, Kuala Lumpur 56000, Malaysia; suzana.shahar@ukm.edu.my

**Keywords:** cognitive frailty, elderly, Malaysia, health care utilization, comorbidities, out-of-pocket payment

## Abstract

Background: Cognitive frailty (CF) as a potential risk factor for dementia, functional disability, poor quality of life, and mortality. The aim of this study was to explore the health care-related utilization and out-of-pocket (OOP) expenditures, sociodemographic characteristics, and comorbidities among elderly Malaysians with CF. Methods: A cross-sectional study targeting elderly Malaysian aged ≥65 years was conducted. The study included all participants of the fourth phase of the Malaysian representative Long-Term-Research-Grant-Scheme Towards-Useful-Aging (LRGS-TUA) community-based study. A structured and validated interview questionnaire was used. Results: In total, 1006 elderly were interviewed, with a 66.18% response rate. Only 730 respondents met the inclusion criteria. Of the eligible respondents, the CF prevalence was 4.5%. Around 60.6% of the participants with CF had utilized outpatient care at government clinics within the past 6 months. The estimated mean total OOP payments for CF during the past 6 months was 84 Malaysian Ringgit (RM) (SD 96.0). Conclusions: CF among elderly Malaysians is within the internationally recognized range of prevalence. The OOP payments for seeking health care among CF elderly are not different from that of other elderly categories. There is a high possibility of underutilization of the health care services of CF cases while they are still needy.

## 1. Introduction

According to international reports, human life expectancy increased from 61.7 years to 71.8 years between 1980 and 2015 [1,2]. The percentage of elderly people is increasing faster than that of any other age group [1]. Currently, this age group is approaching 13% of the global population, with a 3% annual growth rate [1]. Additionally, the expected global number of people aged ≥60 years will increase from 900 million to 2 billion between 2015 and 2050 [1,3]. Consequently, geriatric-related illnesses are expected to increase, leading to an urgent need to build comprehensive health care services that are appropriate for elderly needs [4]. This increase can be attributed to health care services advancement and increased access to it [4].

Within the context of Malaysia, life expectancy of the population increased from 71.3 years between 1990 and 1995 to 75.5 years between 2015 and 2020; it is expected that Malaysians’ life expectancy will reach 80.1 years between 2045 and 2050 [1]. According to the Malaysian Department of statistics, it is expected that the population size will reach 41.5 million in the year 2040 [5]. Malaysia is expected to transform into an aging nation; the Malaysian department of statistics reported that elderly people aged 60 and above constituted around 10.7% of the total population in the year 2020 [6]. It is expected that elderly people aged 60 and above will become 14.5% of the population in the year 2040 [5]. The prevalence of successful healthy aging among older people in Malaysia, setting aside any chronic diseases and mental dysfunctions is 13.8% [7].

According to the Malaysia National Health Accounts-Health Expenditure Report 1997–2019, Malaysia had different financing sources for health-related expenditures, including public and private sector in the year 2019 [8]. Among these financing sources, the Ministry of Health spent around RM 28,860 million (45% share of total health expenditure—THE), private household OOP spending was around RM 22,492 million (35% share of the THE), private insurance spent around RM 4889 million (8% share of the THE) and non-health insurance corporations spent around RM 2117 million (3% share of the THE). Additionally, the Ministry of Education spent around RM 1579 million (2% share of the THE) [8].

More recently, there has been concern regarding the occurrence of double geriatric syndromes, i.e., cognitive frailty, as a potential risk factor for dementia, functional disability, poor quality of life, and mortality [9,10]. In addition to physical and psychosocial frailty, cognitive frailty (CF) is considered one of the subtypes of frailty syndromes [10,11,12]. It is characterized by reduced cognitive reserve for resisting cognitive decline or impairment [13,14]. It is recognized as a heterogeneous cognitive condition that results from physical frailty and not from a known neurodegenerative disorder [9,15]. Cognitive frailty is not recognized as a disease, but rather is considered a syndrome [13]. It is defined as a heterogeneous clinical manifestation characterized by the simultaneous presence of both physical frailty (also known as the Fried phenotype and Cardiovascular Health Study [CHS] frailty index) and cognitive impairment as defined by a Clinical Dementia Rating (CDR) of 0.5, without Alzheimer’s disease or dementia or any other brain disease that can lead to dementia [13].

As an elderly-related syndrome, CF and other types of frailty are considered as a health care cost driver for patients, families, and health care systems [16]. This cost varies from one country to another depending on the health care system components and utilization patterns of elderly patients [16]. Health care-driven costs can be classified as direct and indirect; direct costs result from the direct utilization of services, while indirect costs result from the productivity losses of the patients and their companions when seeking health care, and from lost quality-adjusted life years (QALYs) [17]. It is well known that Out-of-Pocket (OOP) payments are crucial parts of the direct cost of health care services from the patient’s perspective. OOP payments are considered important causes of financial catastrophes to patients and families. The identification of OOP payments plays an important role in recognizing the costs that might be the cause of the patient’s financial barriers to seeking health care [18,19].

The present study aimed to identify the health care utilization patterns, OOP expenditures, sociodemographic characteristics, and comorbidities among elderly Malaysians with cognitive frailty.

## 2. Materials and Methods

This was a cross-sectional study conducted between March 2019 and November 2019. The target population included all elderly Malaysian citizens aged 65 years and above. The study included the fourth phase participants of the Malaysian representative LRGS TUA (Long Term Research Grant Scheme—Towards Useful Aging) longitudinal community-based study [20] as a representative to the Malaysian community. The LRGS TUA study included a total of 1520 participants, a multistage systematic random sampling technique was followed for the selection of cases out of the Malaysian elderly population. The purpose of following this sampling technique was to get a representative sample of community dwelling elderly Malaysians comprising the three main ethnic groups in the country. The inclusion criteria were elderly Malaysian citizens aged 65 years old or above. While the exclusion criteria were elderly participants who had: physical disability (i.e., bedridden, wheelchair user); terminal illnesses; psychiatric problems (i.e., dementia and Alzheimer’s disease); a history of alcohol or drug abuse. These criteria were considered exclusion criteria based on the definition of cognitive frailty by Kelaiditi et al. [13]. Patient approval and informed consent were obtained; each patient was given a full explanation of the aim of the study and its processes. In the present study, cases were identified based on the definition of cognitive frailty by Kelaiditi et al. [13]. Non-respondents were identified as LRGS TUA third-phase study participants who failed to attend their interview appointments or who had passed away.

The sample size calculation was done by using the Cochran (1963) formula for prevalence studies (as shown below). The sample size calculation criteria were: 95% confidence level, 5% confidence interval, 50% population proportion, and population size of 3 million elderly people of Malaysia.

$$N=\frac{z^{2}\times P\left(1-P\right)}{X^{2}}$$

According to the literature, the prevalence of CF was ranged between 4% and 8% of the elderly population and depending on the sample specifications. The larger sample size was considered in the current study. So, the sample size for the current study was 369 + 20% drop out = 443 participants. The final sample size of the study was 1520 participants (based on the currently available participants of the LRG TUA study).

Data were collected through a structured interview questionnaire consisting of three data collection tools that have been previously published elsewhere: the Fried criteria of frailty [21]; the Clinical Dementia Rating (CDR) test [22]; and the inpatient and outpatient health care utilization and payments assessment tool [23]. These three tools were used for identifying participants with cognitive frailty, other frailty, pre-frailty, and robust elderly participants.

The first part of the questionnaire included five questions based on the Fried criteria [21]. Based on those findings, the participants were classified into three categories: Not frail (or, robust) (0 points), pre-frail (1–2 points), or frail (3–5 points). The second part of the questionnaire included the psychological assessment test (CDR) [22]. Based on this test, the participants were classified into five categories (CDR = 0, 0.5, 1, 2, 3). According to Hughes, et al. [24], CDR scale results are: CDR = 0 for normal mental patients (no dementia); CDR = 0.5 for questionable stage (very mild dementia); CDR = 1 for mild impairment (mild dementia); CDR = 2 for moderate impairment (moderate dementia); CDR = 3 for sever impairment (severe dementia). The CDR test is considered to have high predictability of the progression to Alzheimer’s disease in patients with mild cognitive impairment [25]. Accordingly, the participants were diagnosed with cognitive frailty when they had Fried scores of 3–5 and a CDR score of 0.5 at the same time [13]. The third part of the questionnaire involved assessment of the participant’s medical history, covering the previous year’s inpatient hospitalization and the last 6 months of outpatient care that was designed, validated, and used by Aljunid, et al. [23]. The data were collected at the primary health care facilities during 2019. Furthermore, the questionnaire included assessment of the current morbidities among the study participants as a score. The morbidity score was based on the total count of health related problems out of 24 most common problems among the elderly. Lastly, the questionnaire comprised the disability assessment schedule 2.0 questions (WHODAS 2.0) developed by the World Health Organization [26]. With reference to Andrews, Kemp [27], this assessment schedule (WHODAS 2.0) was used as an effective test for the identification of frailty exclusion cases due to disabilities, as required by Kelaiditi et al. [13] criteria for the definition of cases with cognitive frailty.

The study used the Statistical Package of Social Science (windows version 20, SPSS, Chicago, IL, USA). To assess the significant relationships among the study findings, descriptive statistics were performed, cross-tabulations, general scores, Chi-square test (χ^2^), One way anova, and The post hoc Bonferroni test.

## 3. Results

Table 1 presents the participants’ sociodemographic characteristics. A total of 1006 participants of the fourth phase of the LRGS TUA study were interviewed, with a 66.18% response rate. By applying the exclusion criteria of the study sample, 204 responding (20.3%) participants were excluded due to high disability scores (more than 9) based on the Kelaiditi et al. [13] definition of CF syndrome. Fried’s criteria and the CDR test could be applied to 730 respondents who fulfilled the inclusion criteria of the study. The participants were mainly of Malay ethnicity, followed by those of Chinese and Indian ethnicity (64.5%, 31.6%, and 3.9%, respectively). There were 4.5% (*n* = 33) of the participants with cognitive frailty syndrome, while 3.6% (*n* = 26) of the participants had other types of frailty. The participants with CF were from Kelantan, Selangor, and Perak (69.6%, 15.2%, and 15.2%, respectively). There were no cognitive frailty cases from Johor. Participants with CF were distributed almost evenly among the age groups between 65 to 80 years old (65–70 years, 71–75 years, and 76–80 years) with total participants of 11, 9, and 11, respectively. Of the cognitive frailty cases, 72.7% (*n* = 24) had school-level education; the majority of CF participants (97%, *n* = 32) were unemployed and were categorized under the low-income group.

Table 2 shows the participants’ comorbidities. Hypertension, high cholesterol levels, vision problems, and diabetes mellitus were the most common chronic diseases (54.9%, 52.2%, 35.8%, and 27.7%, respectively). Notably, hypertension and higher blood cholesterol levels were more prevalent (73.1% and 69.2%, respectively) among the participants with other-type frailty compared to the not frail, pre-frail, and CF participants. With regard to the number of comorbidities among the study participants, the most common comorbidity scores among all elderly participants were 4, 3, and 2 comorbidities with 19.7%, 15.6%, and 14.8% of the participants, respectively. The most common comorbidity scores among CF participants were 4, 6, and 5 comorbidities with 30.3%, 24.2%, and 15.2% of the CF participants, respectively. One-way ANOVA tests were performed to examine the presence of significant differences among the study participants related to comorbidities and frailty status. A statistically significant relationship was observed among the participants (F-value = 9.302, *p*-value < 0.001). The post hoc Bonferroni test revealed a statistically significant difference in the comorbidity scores between CF patients and both robust and pre-frail participants. The CF participants had comorbidities more than both the robust and pre-frail participants (*p*-value < 0.001 and *p*-value = 0.013, respectively).

With regard to the current disability status among the study participants, the disability score was calculated. The mean disability scores among the robust, prefrail, other frailties, and CF participants were 2.52, 3.03, 4.23, and 5.3, respectively. One-way ANOVA tests were performed to examine the presence of significant differences among the various categories of the study participants related to disability scores. A statistically significant difference was observed among the participants related to the degree of disability (F-value = 10.766; *p*-value < 0.001). The post hoc Bonferroni test has revealed a statistically significant difference in the disability score between CF and both robust and pre-frail participants. The CF participants had a higher disability score than that for both the robust and pre-frail participants (*p*-value < 0.001 and *p*-value < 0.001, respectively). So, in our study there is a statistically significant association between cognitive frailty and disability.

Table 3 shows the participants’ outpatient health care utilization during the past 6 months. In total, 81.8% (*n* = 27) of the CF participants reported a mean of 2.26 visits (with a maximum of 5 visits) to the health care facilities for seeking treatment during the last 6 months. One-way analysis of variance (ANOVA) was conducted to examine the presence of significant differences between the participants with CF and the other participants, namely: not frail, pre-frail, and other frailties. No statistically significant differences were found in the number of visits among all participants (F-value = 2.451, *p*-value = 0.062).

During the past 6 months, the participants with CF utilized outpatient care at government clinics (60.6%), government hospitals (21.2%), and private clinics (21.2%). None of them sought outpatient care at private hospitals, traditional medicine healers, or alternative health care. Of the CF participants, around half (45.4%; *n* = 15) visited government clinics more than once (with a maximum of 3 visits). Only 18.1% (*n* = 6) of the participants with CF visited outpatient clinics at government hospitals more than once (with a maximum of 3 visits).

Notably, the participants with CF did not utilize inpatient hospital care services: only 3% of them (*n* = 1) were admitted to government hospitals during the previous year. The diagnosis for admission was myocardial infarction. No participant with CF was admitted to a private hospital. The study found that transport cost was accounting for around 20% of the total OOP payments during health care seeking among CF and other elderly people in Malaysia.

**Figure 1:**
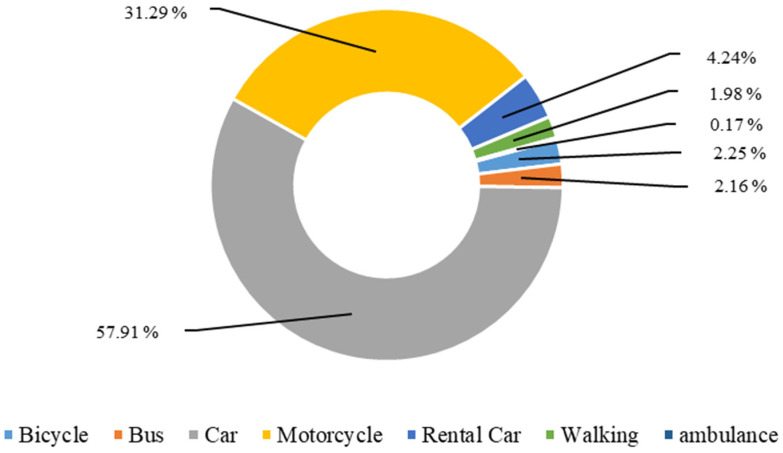
Type of vehicles used for transport to outpatient health care facilities during the past six months.

Figure 1 shows the type of vehicles used for transport to outpatient health care facilities during the past 6 months. The most common type of vehicle used among the CF elderly and other elderly categories (not frail, pre-frail, other frailties) were private cars and motorcycles, while public transport was not commonly used.

**Figure 2:**
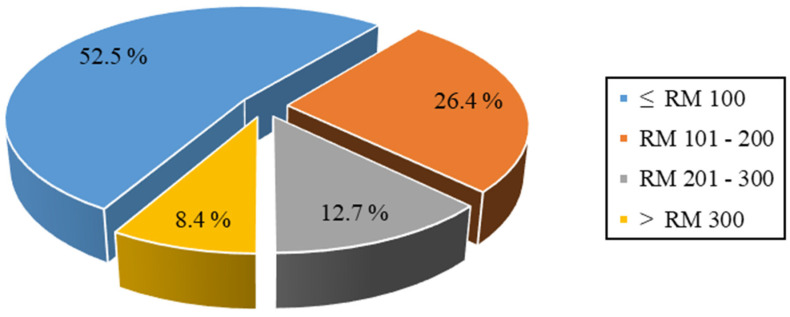
Total out-of-pocket expenditure for seeking of care during past six months; *RM, Malaysian Ringgit*.

Figure 2 shows the participants’ categories of the total OOP expenditure for seeking care during the past 6 months among elderly Malaysians. More than half of the participants (52.5%) spent less than RM 100 for seeking health care services out of their pockets, while 26.4%, 12.7%, and 8.4% of the participants spent RM 101–200, RM 201–300, and >RM 300 for the same purpose, respectively. The presence of significant OOP payment differences between the participants with CF and the other groups of participants was examined using the chi-square test. No statistically significant difference was observed in the total OOP payments between all groups (Sig = 0.085). The estimated mean total OOP payments for seeking health care every 6 months among the whole sample was RM 102.5. The higher mean OOP expenditure during the past 6 months involved special food treatments (RM 80), followed by medication charges (RM 59).

The mean total OOP payment for 6 months of care among elderly Malaysians with CF was around RM 84 (SD = 96), while robust, pre-frail, and participants with other frailties were with total OOP payments of RM 106 (SD = 132.5), RM 103 (SD = 116.1), RM 90.8 (SD = 64), respectively. One way ANOVA tests were conducted to examine the presence of statistically significant differences between CF participants and other groups of elderly participants in the OOP payments. There were no statistically significant differences in the OOP payments for seeking care during the past 6 months among the CF, other frailty types, pre-frail, and robust participants (F-value = 0.397, *p*-value = 0.755).

**Figure 3:**
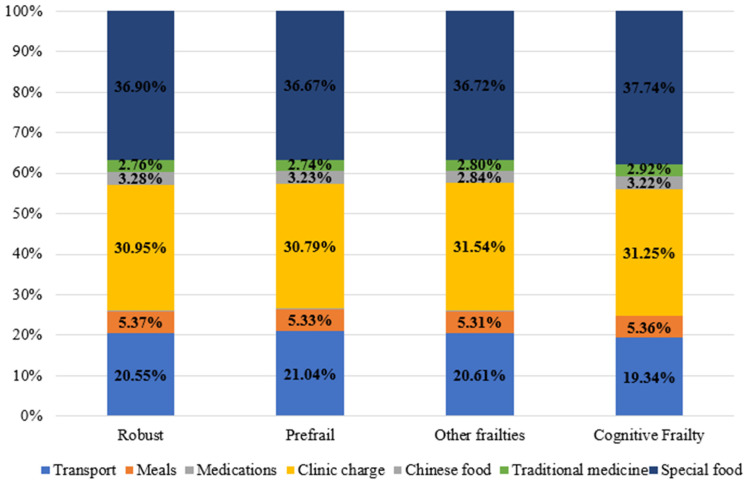
Type and percent of out-of-pocket payments for care during past six months.

Figure 3 shows the participants’ type and percentage of OOP expenditure for seeking health care during the past 6 months. Among the whole sample, the biggest OOP payments during health care seeking were for special food costs (37%) and clinic charges (31.1%). No statistically significant differences were observed in the OOP payments for transport, meals, drug charges, clinic charges, traditional medicine, special food, and the total OOP payments for seeking care during the past 6 months among the CF, other frailty types, pre-frail, and robust participants. There was a significant difference only in the OOP payments for Chinese medicine among the participants (F-value = 5.062, *p*-value = 0.002). The post hoc Bonferroni test has revealed a statistically significant difference in the OOP payments for Chinese medicine between the robust, CF, and pre-frail participants only. The robust participants used Chinese medicine more than both the CF and pre-frail participants (*p*-value = 0.022 and *p*-value = 0.009, respectively).

## 4. Discussion

This study was conducted among Malaysian citizens aged 65 years and above. The participants of the study were from the LRGS TUA community-based study as a representative of the Malaysian community. The inclusion criteria were all Malaysian citizens aged 65 years and above, having no physical disability, no terminal illness, no psychiatric problem, and no history of alcohol or drug abuse. The study was designed to assess the participants’ characteristics, healthcare utilization patterns, and out-of-pocket payments for health care among elderly.

The current study reported that elderly aged 65 to 70 years, 71 to 75 years, 76 to 80 years, and 81 years or above were around 43.3%, 32.3%, 17.7%, and 6.7%, respectively. According to the Malaysian Department of Statistics (2021), elderly aged 65 to 69 years, 70 to 74 years, 75 to 79 years, and 80 years or above were representing 39.3%, 27.5%, 15.3%, and 17.9%, respectively [28]. With little variation in the age groups, these findings indicate that the current study sample is almost similar to the distribution of elderly ages among the Malaysian community.

Regarding the CF syndrome, the prevalence of CF syndrome was 4.5% among the study participants. This finding was consistent with several studies around the world; these studies included Roppolo, Mulasso [29], Ma, Zhang [30], Panza, Lozupone [31], Arai, Satake [32], Liu, Han [33], Lee, Kim [34], Aliberti, Cenzer [35], Moon, Huh [36,37], and two recent studies conducted by Jing, Li [38] and Navarro-Pardo, Facal [39].

Additionally, the prevalence of CF in the current study was different from that observed in other studies. These studies were categorized into those with low CF prevalence and studies with high CF prevalence. Among the studies with low CF prevalence, the reported CF prevalence was ranging between 1.2% and 2.6%. These studies include those conducted by Shimada, Makizako [40], Feng, Nyunt [9], Merchant, Chen [41], Solfrizzi, Scafato [42], Shimada, Makizako [43], Chye, Wei [44], Ma, Zhang [45], Kim, Awata [46], Chen, Park [47], Ge, Zhang [48], and the recent study conducted by Ma, Li [49]. It is worth noting that these studies came from other Asian countries (Japan, Singapore, and China).

On the other hand, among the studies with high CF prevalence, these studies were grouped within two different intervals. The first group was with a prevalence interval ranging between 7.2% and 13.3%. Studies within this interval were Montero-Odasso, Barnes [50] with 10.7% CF prevalence; John, Tyas [51] with 12% CF prevalence; Shimada, Lee [52] with 9.8% CF prevalence; Shimada, Lee [52] with 9.6% CF prevalence; Rietman, van der A [53] with 9.2% CF prevalence; Lee, Peng [54] with 8.6% CF prevalence; Tsutsumimoto, Doi [55] with 12% CF prevalence and its follow up after two years by Tsutsumimoto, Doi [56] with 11.2%; Liu, Chen [57] with 13.3% CF prevalence; Majnarić, Bekić [58] with 8% CF prevalence; Brigola, Ottaviani [59] with 13% CF prevalence; Katayama, Lee [60] with 10.7% CF prevalence; and Xie, Ma [61] with 7.19% CF prevalence. Among the expected justifications for changes in CF prevalence are: Firstly, these studies came from different countries on different continents. It is well known that the age distribution and comorbidities among the elderly from different countries are not the same. Secondly, the use of different CF identification tools is expected to cause variations in the reported CF prevalence. Thirdly, the methodology for case selection was different, this can be an additional factor for changes in the CF prevalence among the studies. Lastly, the studies were conducted in different years starting from 2016 until 2021. This could affect the CF prevalence findings.

Additionally, the second group with high CF prevalence intervals were ranging between 22% and 35.7%. Studies within this interval were Delrieu, Andrieu [62] with 22% CF prevalence; Hao, Dong [63] with 50% CF prevalence; Esteban-Cornejo, Cabanas-Sánchez [64] with 22.6% CF prevalence; Kwan, Leung [65] with 35.7% CF prevalence; and Seesen, Sirikul [66] with 28.72% CF prevalence. This variation could be due to several factors including using different CF assessment tools, variations among the participants’ sociodemographic characteristics, and variations among the participants’ comorbidities [31,67]. Nevertheless, Roppolo, Mulasso [29] used different assessment tools for CF syndrome (MMSE and CHS) and reported the same prevalence of the current study.

Mostly, frailty detection tools, sociodemographic and comorbidity characteristics of the study samples are considered as the most contributing factors for such prevalence variations [31]. Furthermore, the CF prevalence of the current study was different from that observed by Delrieu, Andrieu [62]. This variation was due to the summation of frail or pre-frail patients within the same group in that study [62]. Such action is expected to produce a higher CF prevalence compared to the current study. In conclusion, the prevalence of CF syndrome among Malaysians is considered within the international range. The current CF prevalence finding was consistent with that reported in the review articles by Sugimoto, Sakurai [67] and Arai, Satake [32]. Additionally, as a community-based study, the current findings are also consistent with the reported CF prevalence among the studies following the same methodology of the sample, more specifically, the studies following community-based methodology as stated by Panza, Lozupone [31].

Regarding the CF syndrome findings of the current study, CF syndrome prevalence was more common among married, Malay, males, with school level education or below, not having any work, with income less than RM 1000 per month, and more commonly from the state of Kelantan. More specifically, the current study found that CF was more common among males with 66.7% compared to females; this finding was consistent with Rietman, van der A [53] study (CF males were 67.4%). Regarding the marital status of participants, it was found that 42.4% of the CF participants were either widowed or divorced. It is known that marital status can be considered an important risk for the development of CF syndrome [30,68]. The study findings revealed that 72.7% of the CF patients had low level education (school-level); this could negatively affect the patients through reduced knowledge and interest on the value of a healthy lifestyle, health promotion and disease prevention importance to the elderly. Among the literature, it was reported that the CF prevalence was relatively higher among illiterate participants [30]. This was consistent with the current study findings.

The majority of CF participants (97%) were unemployed and categorized under the low-income group; this was consistent with the study findings of AB and Juni [69]. Additionally, it is well known that the elderly are recognized as a more vulnerable group of people with higher expenditures [70]. Furthermore, among the literature, it was reported that CF prevalence was relatively higher among low-income participants [30]. It is worth mentioning that the elderly with higher income levels can spend more on health care services and gain more health [71]. Likewise, the elderly with lower income levels spend less on health care services and are expected to gain less health compared to others. Additionally, health-related expenditures are important causes of financial catastrophes to patients and families [72,73]. It is worth mentioning that age is considered a significant influencing factor for higher health care related OOP payments [74,75]. So, the elderly had a higher probability to suffer from catastrophic health expenditures [75]. As the majority of the CF patients were under the low income category, they are expected to afford health-related payments more than 10% of their ability to pay as identified by Cylus, Thomson [76] for the definition of catastrophic expenditures. This made them within the category who suffer from financial catastrophes while seeking health care. For all that is mentioned above, it is expected to reduce the elderly’s willingness to seek health care and the health care services utilization rates.

Regarding the comorbidity score of participants, it was observed that CF participants had higher comorbidity scores compared to robust and pre-frail participants. Among the literature, it was reported that comorbidities are more common among CF patients [30,46,57]. It is worth mentioning that increased morbidities are associated with more health care utilization and higher burden [70,77,78]. This indicates consistency between the current study findings and other studies concerned with comorbidity.

As noted in the literature, comorbidities increase the risk of health care related catastrophic expenditures among the elderly [79]. Additionally, chronic diseases increase the probability of health related OOP payments [80]. More specifically, frailty syndromes increase the risk of catastrophic health expenditures among single elderly with morbidities [68]. Such payments need to be assessed carefully because they affect negatively the necessary life expenses [81]. These can be considered as additional important risk factors that need to be addressed as soon as possible to reduce the CF prevalence and its burden among Malaysians. Working on managing and reducing age related morbidities among CF elderly is crucial for public health experts to handle [82].

Regarding the disability score of the study participants, it was observed that CF patients had higher disability scores compared to both robust and pre-frail participants with a statistically significant difference. This indicates that the elderly with CF syndrome are associated with an increased disability compared to others. In the literature, it is well known that CF is considered a potential risk factor for functional disability [9,10]. The current study findings are consistent with several recent studies; these studies revealed that CF syndrome is a significant risk factor for disability among the elderly [35,47,56,61]. It is well known that CF syndrome is considered as a transitional stage between healthy aging and disability [11,83,84,85,86,87]. Additionally, this syndrome is characterized by reduced reserve to resist any cognitive decline or impairment [13,14]. These facts indicate a high possibility for rapid change of CF patient’s health status into the disability category of people due to health deterioration. All these findings strengthen the premise that CF prevalence among elderly Malaysians could be higher than the currently observed one as many patients might become disabled within a short period of time due to unmanaged CF syndrome. For all points mentioned above, it seems that there is underutilization of the health care services by CF patients, as they have higher scores of morbidities and disability; they might become unable to seek health care services when needed. Furthermore, it is not clear how big the prevalence of CF Syndrome is among the non-respondents in the current study (*n* = 514). This indicates that there is a possibility to find additional deteriorated CF cases among them which made them unable to attend the interview of the study due to health-related problems. So, we can conclude that many cases with CF syndrome among elderly Malaysians are expected to change rapidly into the disability category of the elderly as they are not getting proper health care services. This can lead us to a premise that CF syndrome is with a higher burden among elderly Malaysians. As CF syndrome increases the possibility of getting disabilities, such a condition will increase the elderly’s dependency on others [88]. It is worth noting that elderly dependence increases the burden on individuals, families, and health care systems [89]. In a cost wise expression, it also suggests the presence of further additional undetected health burdens of CF syndrome on patients, families and health care systems. Such findings necessitate immediate action to control this syndrome.

Here, around 82% of the CF participants reported utilizing outpatient care facilities during the last 6 months. This is consistent with findings of García-Nogueras, Aranda-Reneo [90] and Fairhall, Sherrington [91] studies on frailty syndrome; while the number of visits to health care facilities in both studies mentioned above was much higher than that observed in the current study.

The hospitalization rate among all frailty types of the present study was much lower than that in several studies [90,91,92,93,94,95,96,97,98,99,100]. Such hospitalization rate differences could be due to the adoption of different health promotion and disease prevention programs, higher income levels, different awareness levels of the importance of seeking health care services, or due to health care systems variations among these countries. This finding may suggest a weak optimistic scenario that elderly Malaysians are probably healthier than those in the other countries, but many other findings of the current study do not support this probability (increased morbidity and disability scores findings).

In contrary, another worse scenario is still strongly possible. This scenario suggests that these low utilization rates among the CF participants could be due to several reasons: Firstly, recall problem or missed reporting of the episodes of health care during the data collection interviews due to mental weaknesses. Secondly, a physical weakness due to higher comorbidities and disabilities that hinders them utilizing health care services. Thirdly, underutilization of the health care services due to lower levels of awareness on the importance of routine health care service attendance for health checkup. Fourthly, due to a lack of specific health programs within the primary health care facilities that focus on elderly needs. Such probabilities need to be addressed carefully. It is worth noting that Hamid, Momtaz [7] reported the prevalence of successful aging among older persons in Malaysia was 13.8%. This prevalence became 10% after four years [20]. This makes it more necessary to take immediate action to control this syndrome through adopting health promotion and disease prevention interventions among the community [85,101].

According to Chang, Cowling [102], the global average OOP expenditure for seeking health care during 2016 was 200.3 United States Dollars (USD) per year. Additionally, the global average OOP expenditure for seeking health care is expected to reach USD 317 in the year 2050; for upper middle-income countries, it is expected to reach USD 425 per year [102]. For Malaysia, the average out-of-pocket health spending per capita for outpatient health care, inpatient health care, oral health care, community pharmacy, domiciliary care, dietary supplements and others was around RM 650.70 in the year 2019 [103]. OOP spending for outpatient care was accounting for around 40.4% of the OOP health spending per capita in Malaysia [103]. The health expenditure per capita for the last 12 months inpatient care among the hospital care users was around RM 964.83 in the year 2019 [103]. Malaysia is classified as an upper middle-income country [104]. The present study shows that elderly Malaysians spend around RM 205 per year for OOP for seeking health care (USD 50). The CF respondents spent OOP around RM 168 (USD 41) per year for seeking health care, while the robust elderly spent OOP around RM 212 (USD 51) per year for seeking health care. However, the reported variation in the total OOP payments between the CF and other elderly categories was not statistically significant. Notably, only one CF patient was admitted to the hospital, while several cases were reported with inpatient episodes from other elderly categories. This could be the reason for the low amount of total OOP payments among the CF group. So, the observed low average OOP payments and costs among CF participants compared to other categories suggests that they are either of two possibilities: Firstly, CF patients are not attending health care services while they are in real need of them; Secondly, CF patients missed reporting the health care episodes during the interview with the enumerators due to mental weakness leading to poor recall. An additional expected reason for low OOP payments among the CF group could be due to non-attendance of many possible CF cases at the interviews during conduction of the study, as they could have deteriorated health status due to increased morbidity and disability scores. These cases are expected to have high amounts of OOP payments that will raise the mean OOP payments among the whole CF category of patients. Furthermore, the current low-cost findings among CF participants might be related to more utilization of the public health care services; it was noticed that 60.6% of CF participants sought health care at governmental hospitals, 21.2% of CF participants sought health care at governmental clinics, 21.2% of CF participants sought health care at private clinics, while none of the CF cases sought health care at private hospitals. It is well known that private health care is considered more expensive compared to public health care services. For Malaysians, it is worth mentioning that Malaysian public health care facilities provide highly subsidized inpatient and outpatient health care services to its citizens [105]. This would affect the total OOP payments of elderly Malaysians seeking health care in general; these high governmental subsidizations can lead to reduced total OOP payments compared to those observed in other countries. The additional justifications for lower Malaysian OOP spendings among CF patients compared to other countries are the lower health care utilization rates of elderly Malaysians, lower gross domestic product per capita compared to European countries, and adoption of different health insurance types among different countries. All can be considered a reasonable justification for such cost variations. Additionally, variations in the sociodemographic and comorbidity characteristics of participants among different studies, the use of different CF syndrome assessment tools, and the use of different methods for participant selection can also be influencing factors that can affect the health care costs for the elderly.

Most likely, the elderly with CF syndrome in Malaysia have low health care costs for a short period of time. This can be followed by a significant increase in cost due to deterioration in health status as a result of the onset of high disability and morbidity burdens. These increased morbidities and disabilities will reduce the elderly’s productivity and increase their dependency on others. It is worth mentioning that CF syndrome increases the risk of dependency [88]. This means that there will be lost or reduced productivity of the patients or family members; this will incur higher health care related costs for all of them [106]. So, elderly dependence is associated with increased OOP payments and burden on individuals, families, and health care systems [89].

Finally, noting that a large percentage of CF participants are from low-income and low-education categories we can conclude that they had lower financial willingness to pay, lower levels of awareness of the importance of health care follow-up, and lower levels of physical fitness to go to health care facilities seeking care when needed. So, integrating health promotion and disease prevention programs among the public health care services is increasingly claimed to be needed for the elderly; these preventive actions will provide benefits, not only for CF patients but also for all elderly Malaysians as well [85,101]. All these benefits can be considered as additional benefits over that gained by the CF elderly.

The limitations of the current study may include some issues. Firstly, the small number of cases diagnosed with CF, which might be resulting from the absence of some CF cases among the non-respondents or because of the small prevalence of this syndrome among elderly Malaysians. To overcome this issue, the study sample size was considered to be three times the calculated sample. Secondly, participants’ recall bias for both the health care utilization patterns and the paid cost during seeking of health care. Work has been done to overcome this issue by re-asking the questions for the respondents in layperson terms and giving them proper time to think before answering, in addition to allowing assistance by companion relatives. Furthermore, using closed-ended questions during the interview, might hinder some important information. This issue was overcome by using a previously validated and used questionnaire. Lastly, samples were not collected from Borneo Island due to various reasons and constraints including time, cost and some of the tools would need validation for usage among older adults in Borneo which have a distinct sociodemographics, local languages and cultures. We are confident that findings from the four selected states in the study are representative of the population of Peninsular Malaysia.

## 5. Conclusions

The prevalence of CF syndrome was 4.5% among the study participants. The CF syndrome prevalence was more common among married, Malay, males, with a school level of education or below, not having any work, with an income less than RM 1000 per month, and more commonly from the state of Kelantan. The current findings are consistent with the reported CF prevalence figures among international studies following community based methodologies. It was observed that CF participants had higher comorbidity and disability scores. This indicates that CF syndrome is associated with an increased probability of disability compared to other categories. So, it is expected that elderly Malaysians with CF syndrome are moving into the disability stage of the elderly. As CF syndrome increases the possibility of getting disabilities, such conditions will increase the elderly’s dependency on others leading to an increased burden on patients and families and increased total cost. Based on the current study findings, the premise of higher burden among CF patients in Malaysia can be highly accepted.

## Figures and Tables

**Table 1:** Sociodemographic Characteristics of the Study Participants.

		Not Frail *n* (%) *	Pre-Frail *n* (%) *	Other Frailty *n* (%) *	CF *n* (%) *	Total *n* (%)
State	Perak	41 (5.6)	134 (18.4)	2 (0.3)	5 (0.7)	182 (24.9)
	Selangor	30 (4.1)	154 (21.1)	3 (0.4)	5 (0.7)	192 (26.3)
	Kelantan	29 (4.0)	177 (24.2)	17 (2.3)	23 (3.2)	246 (33.7)
	Johor	36 (4.9)	70 (9.6)	4 (0.5)	-	110 (15.1)
	Total	136 (18.6)	535 (73.3)	26 (3.6)	33 (4.5)	730 (100)
Gender	Male	44 (6)	270 (37)	17 (2.3)	22 (3)	353 (48.4)
	Female	92 (12.6)	265 (36.3)	9 (1.2)	11 (1.5)	377 (51.6)
Ethnicity	Malay	62 (8.5)	361 (49.5)	22 (3)	26 (3.6)	471 (64.5)
	Chinese	69 (9.5)	156 (21.4)	2 (0.3)	4 (0.5)	231 (31.6)
	Indian	5 (0.7)	18 (2.5)	2 (0.3)	3 (0.4)	28 (3.9)
Marital status	Single	-	9 (1.2)	-	-	9 (1.2)
	Married	95 (13)	367 (50.3)	19 (2.6)	19 (2.6)	500 (68.5)
	Others	41 (5.6)	159 (21.8)	7 (1)	14 (1.9)	221 (30.3)
Age	65–70 Years	83 (11.4)	213 (29.2)	9 (1.2)	11 (1.5)	316 (43.3)
	71–75 Years	37 (5.1)	179 (24.5)	11 (1.5)	9 (1.2)	236 (32.3)
	76–80 Years	15 (2.1)	101 (13.8)	2 (0.3)	11 (1.5)	129 (17.7)
	81–85 Years	1 (0.1)	38 (5.2)	3 (0.4)	2 (0.3)	44 (6)
	>85 Years	-	4 (0.5)	1 (0.1)	-	5 (0.7)
Education level	No Education	21 (2.9)	77 (10.5)	1 (0.1)	7 (1)	106 (14.5)
	School level	113 (15.5)	431 (59)	25 (3.4)	24 (3.3)	593 (81.2)
	Higher education	2 (0.3)	26 (3.6)	-	-	28 (3.9)
	Others	-	1 (0.1)	-	2 (0.3)	3 (0.4)
Current job	Not working	110 (15.1)	448 (61.4)	23 (3.2)	32 (4.4)	613 (84.0)
	Working	26 (3.6)	87 (11.9)	3 (0.4)	1 (0.1)	117 (16.0)
Monthly income	<RM 700	60 (8.2)	200 (27.4)	11 (1.5)	19 (2.6)	290 (39.7)
	RM 701–1400	30 (4.1)	128 (17.5)	11 (1.5)	8 (1.1)	177 (24.2)
	RM 1401–2100	20 (2.7)	103 (14.1)	2 (0.3)	5 (0.7)	130 (17.8)
	RM 2101–2800	4 (0.5)	30 (4.1)	1 (0.1)	-	35 (4.8)
	RM 2801–3500	13 (1.8)	33 (4.5)	-	-	46 (6.3)
	>RM 3500	9 (1.2)	41 (5.6)	1 (0.1)	1 (0.1)	52 (7.1)

* Percentage out of total sample.

**Table 2:** Comorbidities Among the Study Participants.

	Not Frail *n* (%) †	Pre-Frail *n* (%) †	Other Frailty *n* (%) †	CF *n* (%) †	Total *n* (%)	*p*-Value
Hypertension	68 (50)	291 (54.4)	19 (73.1)	23 (69.7)	401 (54.9)	0.051
High cholesterol	63 (46.3)	278 (52)	18 (69.2)	22 (66.7)	381 (52.2)	0.053
Diabetes mellitus	26 (19.1)	156 (29.2)	7 (26.9)	13 (39.4)	202 (27.7)	0.050 *
Stroke	3 (2.2)	21 (3.9)	2 (7.7)	2 (6.1)	28 (3.8)	0.478
Joint pain	46 (33.8)	177 (33.1)	9 (34.6)	16 (48.5)	248 (34)	0.349
Heart disease	9 (6.6)	50 (9.3)	7 (26.9)	3 (9.1)	69 (9.5)	0.014 *
Vision problems	51 (37.5)	191 (35.7)	6 (23.1)	13 (39.4)	261 (35.8)	0.534
hearing problems	17 (12.5)	70 (13.1)	6 (23.1)	7 (21.2)	100 (13.7)	0.279
Renal failure	2 (1.5)	16 (3)	4 (15.4)	4 (12.1)	26 (3.6)	<0.001 *
Chronic lung disease	3 (2.2)	5 (0.9)	-	-	8 (1.1)	0.507
Constipation	15 (11)	79 (14.8)	10 (38.5)	10 (30.3)	114 (15.6)	<0.001 *
Gastric ulcer	10 (7.4)	77 (14.4)	6 (23.1)	4 (12.1)	97 (13.3)	0.074
Cancer	4 (2.9)	15 (2.8)	-	-	19 (2.6)	0.632
Urinary problems	9 (6.6)	70 (13.1)	7 (26.9)	9 (27.3)	95 (13)	0.002 *

* Statistically significant; † Percentage out of subgroups.

**Table 3:** Outpatient health care utilization findings during the past six months.

	Not Frail *n* (%) *	Pre-Frail *n* (%) *	Other Frailty *n* (%) *	CF *n* (%) *	Total *n* (%)
Seek any treatment as an outpatient for the illness suffered in the last six months					
No	32 (4.4)	111 (15.2)	3 (0.4)	6 (0.8)	152 (20.8)
Yes	104 (14.2)	424 (58.1)	23 (3.2)	27 (3.7)	578 (79.2)
The facilities the patient sought treatment in the last six months					
Government clinic	67 (9.2)	275 (37.7)	15 (2.1)	20 (2.7)	377 (51.6)
Government hospital	21 (2.9)	126 (17.3)	7 (1)	7 (1)	161 (22.1)
Private clinic	24 (3.3)	68 (9.3)	3 (0.4)	7 (1)	102 (14)
Times got treatment in government clinic during the last 6 months					
One visit	34 (4.7)	93 (12.7)	8 (1.1)	5 (0.7)	140 (19.2)
Two visits	27 (3.7)	126 (17.3)	4 (0.5)	11 (1.5)	168 (23.0)
Three visits	7 (1)	46 (6.3)	3 (0.4)	4 (0.5)	60 (8.2)
Times got treatment in government hospital during the last 6 months					
One visit	12 (1.6)	64 (8.8)	3 (0.4)	1 (0.1)	80 (11)
Two visits	6 (0.8)	44 (6)	5 (0.7)	4 (0.5)	59 (8.1)
Three visits	2 (0.3)	18 (2.5)	0 (0)	2 (0.3)	22 (3)
Times got treatment in private clinic during the last 6 months					
Non	112 (15.3)	463 (63.4)	23 (3.2)	26 (3.6)	624 (85.5)
One visit	17 (2.3)	51 (7)	3 (0.4)	7 (1)	78 (10.7)

* Percentage out of total sample.

## Data Availability

Supporting data of the study can be accessed by contacting the corresponding author and after approval from the National University of Malaysia—Faculty of Medicine for the data to be accessed.

## Abbreviations

CDR, Clinical Dementia Rating test; LRGS TUA, The Malaysian longitudinal community based study (Long Term Research Grant Scheme—Towards Useful Aging; OOP, Out-of-pocket; RM, Malaysian ringgit; UKM, Universiti Kebangsaan Malaysia (The National University of Malaysia); USD, United States dollar; USM, University of Science Malaysia.
